# Cervical sagittal alignment and its impact on degenerative cervical myelopathy outcomes

**DOI:** 10.1016/j.bas.2025.105862

**Published:** 2025-11-03

**Authors:** Granit Molliqaj, Alexandre Lavé, Michele Da Broi, Leonardo Anselmi, Aria Nouri, Pierre-Pascal Girod, Renato Gondar, Karl Schaller, Enrico Tessitore

**Affiliations:** Department of Neurosurgery, University of Geneva, Geneva, Switzerland

**Keywords:** Cervical sagittal alignment, Deformity, Degenerative cervical myelopathy, Cord compression, Spinal surgery

## Abstract

**Introduction:**

Degenerative cervical myelopathy (DCM) is a progressive spinal cord disorder driven by static compression and dynamic instability. Cervical sagittal alignment has recently emerged as a potential factor influencing both pathogenesis and surgical outcomes.

**Research question:**

This review synthesizes current evidence on alignment parameters and their clinical relevance in DCM management, with the primary aim of guiding surgical decision-making.

**Material and methods:**

A narrative literature review was performed, analyzing radiological parameters of cervical alignment, their normative thresholds, and associations with functional outcomes. Both cranio-cervical and subaxial metrics were examined, alongside emerging global alignment concepts.

**Results:**

Key parameters include C2–C7 sagittal vertical axis (SVA), cervical lordosis (CL), T1 slope (T1S), and the T1S–CL mismatch. Malalignment is generally defined as SVA >40 mm, CL < 15°, or T1S–CL > 20°. Increased SVA and cervical kyphosis correlate with reduced mJOA scores and poorer surgical results. Alignment also informs surgical strategy: anterior approaches are favored in kyphosis or ventral compression, while posterior techniques are preferred in lordotic or neutral spines. Novel measures such as the C2–T1 Pelvic Angle (CTPA) seek to contextualize cervical alignment within global sagittal balance. Evidence further suggests reciprocal cervical adaptations following thoracolumbar correction.

**Discussion and conclusion:**

Cervical sagittal alignment is clinically relevant in DCM pathophysiology and surgical decision-making. While correlations between alignment parameters and outcomes are established, robust evidence defining corrective thresholds remains limited. Prospective studies are required to validate these measures and refine realignment strategies in DCM.

## Highlights

• Cervical sagittal alignment plays a significant role in the pathophysiology and surgical outcomes of degenerative cervical myelopathy (DCM).
• Radiographic parameters such as C2–C7 sagittal vertical axis (SVA), cervical lordosis (CL), and T1 slope (T1S) are associated with functional outcomes and guide surgical planning.
• Kyphotic alignment and increased SVA correlate with worse neurological status and reduced postoperative recovery in DCM patients.
• Surgical approach should be tailored based on alignment, with anterior decompression favored in cases of kyphosis and posterior decompression appropriate in lordotic or neutral spines.
• Emerging global alignment measures, like the C2–T1 pelvic angle (CTPA), aim to integrate cervical parameters into a holistic framework of spinal balance**.**

## Abbreviations

CBVA: Chin-Brow Vertical Angle
CL: Cervical Lordosis
COG: Center of Gravity
COR: Center of Rotation
CTPA: C2–T1 Pelvic Angle
DCM: Degenerative Cervical Myelopathy
EQ-5D: EuroQol-5 Dimension (health status questionnaire)
HRQOL: Health-Related Quality of Life
K-line: A line connecting the midpoints of the spinal canal at C2 and C7 on lateral radiographs
McGS: McGregor Slope
mJOA: Modified Japanese Orthopaedic Association (score)
mK-line: Modified K-line (on MRI)
OPLL: Ossification of the Posterior Longitudinal Ligament
PI-LL: Pelvic Incidence Minus Lumbar Lordosis
SF-36 PCS: Short Form-36 Physical Component Summary
SLS: Slope of Line of Sight
SVA: Sagittal Vertical Axis
T1S: T1 Slope
TIA: Thoracic Inlet Angle
T1S–CL: T1 Slope Minus Cervical Lordosis
VAS: Visual Analog Scale

## 1 Introduction

Degenerative cervical myelopathy (DCM) is an increasingly common presentation in clinical practice, partially due to an increased recognition of the disorder and rise in the aging population (Nouri et al., 2015). DCM develops through direct cord compression from disco-osteophytic formations or ligamentous hypertrophy, often exacerbated by instability-related dynamic injury. However, a more recent and less clearly established concept in the literature is the role of sagittal imbalance on the development of cervical myelopathy. A mix between static and dynamic components could thus play a role and influence the development of cervical myelopathy (Scheer et al., 2013a, 2013b; Ames et al., 2013; Albert and Vacarro, 1998; Buell et al., 2018). The concepts of sagittal alignment in the context of degenerative thoracolumbar disorders are now universally accepted in the spinal surgical community. Generally, malalignment requires correction when substantial, based on radiological parameters such as pelvic incidence, lumbar lordosis, TPA and the SVA plumb line, and when causing progressive deformity, diminished quality of life or onset of neurologic symptoms. Sagittal correction has been shown to improve clinical scores and quality of life of patients (EQ5D, VAS, HRQOL) in patients with thoracolumbar disease (Girod et al., 2023; Kim et al., 2012; Le et al., 2015; Cho et al., 2005; Williamson et al., 2023) As with global sagittal alignment, several radiological parameters for the cervical sagittal alignment are described in the literature. However, there is less evidence concerning their impact on the neurological function or quality of life (Lee et al., 2020). In addition, it is less clear which thresholds and normative values should be targeted for cervical sagittal realignment planning. If sagittal cervical imbalance contributes to DCM, its correction should be included in the surgical strategy. Understanding these parameters is therefore essential not only for predicting neurological outcomes but also for selecting the optimal surgical approach—anterior versus posterior—and for planning the degree of realignment required. In the present review, we have undertaken a comprehensive review of the literature with the aim of addressing the role of cervical alignment in the management of patients with DCM.

## 2 Literature search methodology

This narrative review was conducted using PubMed to identify relevant studies published between 1996 and 2023. Only English-language articles addressing the role of cervical sagittal alignment in degenerative cervical myelopathy were included. We focused on studies reporting relationships between radiographic alignment parameters (e.g., SVA, T1S–CL mismatch, cervical lordosis) and clinical outcomes (mJOA, EQ-5D, neurological recovery) or those guiding surgical approach selection. Case reports and non-English literature were generally excluded unless of historical or clinical relevance.

## 3 Cranio-cervical alignment measurement techniques

**Fig. 1:**
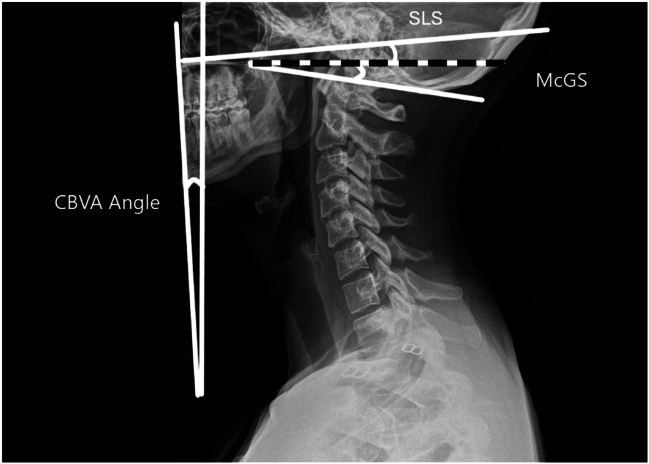
Cranio-Cervical alignment measurement techniques. The chin-brow vertical angle (CBVA, normal range: 5°–17°), the McGregor slope (McGS, −6°–14°), and the slope of line of sight (SLS, −5.1°–18.5°). Unlike conventional C2–C7 alignment parameters, these measurements account for the crucial role of the cranio-cervical junction in maintaining horizontal gaze and spatial orientation.

Several radiographic parameters are frequently described to assess cervical spine alignment, with most of them only considering the C2-C7 segment. However, one must not forget that upper cervical spine also plays an important role in maintenance of horizontal gaze and 3D mobility for orientation in space. Indeed, the cranio-cervical junction contributes around [23°–24.5°] and [10.1°–22.4°] of flexion/extension at the occipitoatlantal and the atlantoaxial joints, respectively, and contributes about a third of rotation (Lopez et al., 2015). A number of measurements techniques exist which take into account the upper cervical spine segment: Horizontal gaze is assessed by the Chin–Brow Vertical Angle (CBVA), defined as the angle between a plane tangential to the glabella and chin and the vertical reference line (normal range −5°–17°); 2) the McGregor Slope (McGS), defined as the angle between a line from the posterosuperior point of the hard palate to the caudal point of the opisthion and the horizontal plane (normal range −6°–14°); and 3) the Slope of Line of Sight (SLS), defined as the angle between the Frankfurt horizontal line and the horizontal plane (normal range −5.1°–18.5°) (Diebo et al., 2016). All these measures can be made on lateral radiographs, illustrated in Fig. 1.

## 4 Subaxial alignment measurement techniques

**Fig. 2:**
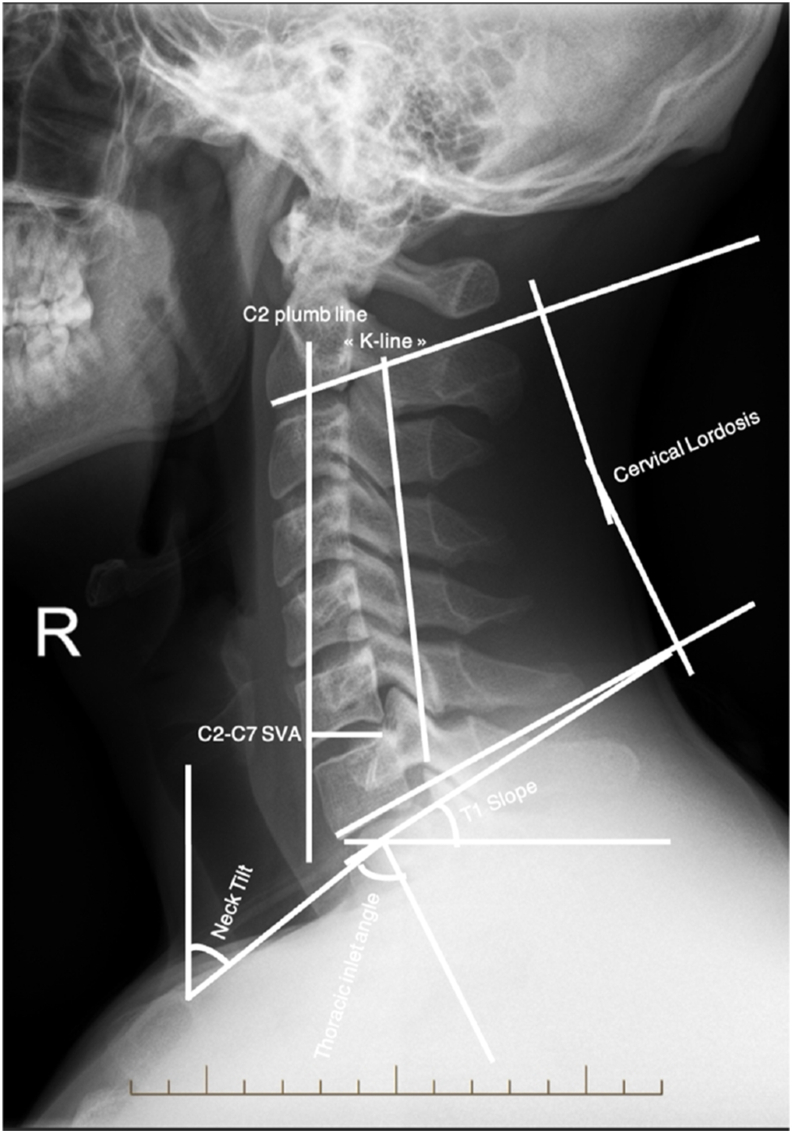
Most frequently reported subaxial cervical sagittal alignment parameters: C2–C7 sagittal vertical axis (SVA), cervical lordosis (CL), T1 slope (T1S), thoracic inlet angle (TIA), K-Line and neck tilt. Although normative values remain debated, commonly accepted thresholds are: T1S < 40°, C2–C7 lordosis >15°, C2–C7 SVA <40 mm, and T1S–CL mismatch <20°.

With regard to the subaxial spine, some authors have tried to apply Dubousset's concept of "cone of economy" in the context of global sagittal alignment at the cervical level by naming it the "cone of kinesis" (Liu et al., 2015). Liu et al. demonstrated in their prospective study of 302 patients that a reduction in the cone of motion (flexion-extension) and a more posterior Center Of Rotation (COR) both correlated with worse neurological function and quality of life (Liu et al., 2015). The main parameters described in the literature for measuring cervical sagittal alignment include the center of gravity (COG), C2-C7 sagittal vertical axis (C2-C7 SVA), cervical lordosis (CL), T1 slope (T1S), thoracic inlet angle (TIA) and neck tilt (Fig. 2). Cobb angles for coronal alignment can complement this assessment. The normal ranges in the cervical segment are still not consensual in the literature but current evidence proposes the following values: T1S < 40°, C2-C7 lordosis >15°, C2-C7 SVA <40 mm, and T1S-CL <20° (Table 1) (Buell et al., 2018). Furthermore, Oe et al. (2015) in a cross-sectional analysis of >500 volunteers over age 50, defined normative thresholds (T1S < 40°, CL > 15°, T1S–CL <20°) and linked deviations with lower EQ-5D scores, highlighting the link between radiographic alignment and quality of life. From a clinical point of view, literature highlighting a correlation between these radiological parameters with disability and myelopathy PROMs are still lacking. However, it has been shown that there is a moderate inverse correlation between kyphosis (C2-C7 SVA) and myelopathy severity (modified Japanese Orthopedic Association score (mJOA)). Kyphotic patients seem to have lower mJOA scores (worst) while lordotic patients seem to have higher scores (Liu et al., 2015; Smith et al., 2013)

**Table 1:** Normative thresholds for cervical sagittal alignment parameters and their clinical significance.

Parameter	Normal Value/Threshold	Description	References
**T1 Slope (T1S)**	<40°	A higher T1S often requires proportionally greater cervical lordosis to maintain horizontal gaze.	Oe et al., 2015 (Oe et al., 2015); Iyer et al., 2016 (Iyer et al., 2016)
**C2–C7 Lordosis (CL)**	>15°	CL < 15° is considered hypolordotic and may indicate cervical imbalance.	Oe et al., 2015 (Oe et al., 2015); Lee et al., 2020 (Lee et al., 2020)
**C2–C7 SVA**	<40 mm	SVA >40 mm is linked to cervical sagittal malalignment and worse neurological outcomes.	Smith et al., 2013 (Smith et al., 2013)
**T1S–CL Mismatch**	<20°	Reflects harmony between cervical lordosis and T1 slope; mismatch >20° is abnormal and associated with sagittal deformity.	Protopsaltis et al., 2018 (Protopsaltis et al., 2018); Oe et al., 2015 (Oe et al., 2015)

**Fig. 3:**
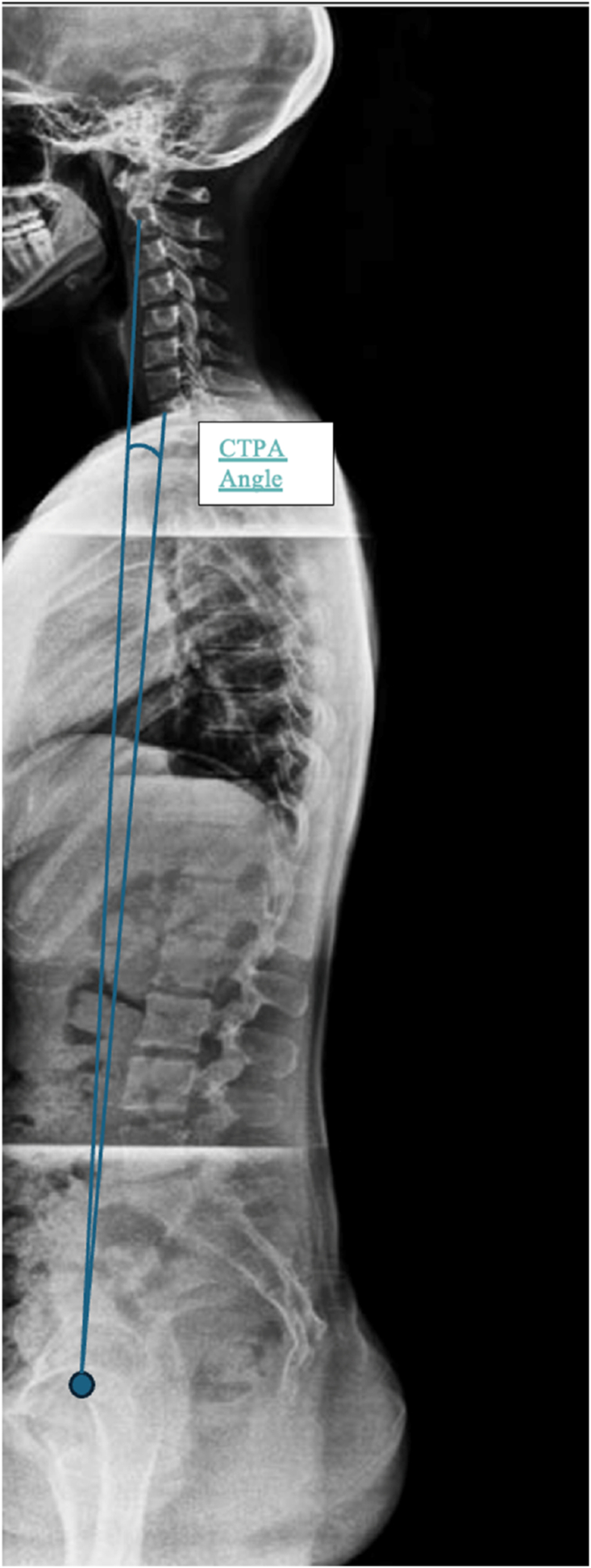
The C2–T1 Pelvic Angle (CTPA) is defined as the angle between a line drawn from the center of the C2 vertebral body to the center of the femoral heads and a line from the femoral heads to the center of T1. This emerging parameter integrates cervicothoracic inclination and pelvic orientation, supporting a more global evaluation of cervico-pelvic sagittal alignment.

Future research is moving towards an integration of such segmental measures into a broader cervico-pelvic balance concept through parameters such as C2-T1 Pelvic Angle (CTPA) (angle formed between a line extending inferiorly from the center of the C2 vertebral body to the center of the femoral heads, and a line extending superiorly from the center of the femoral heads to the center of T1). This angle integrates cervicothoracic inclination (C2-T1) and pelvic orientation (Fig. 3). Compared to the traditionaly used SVA parameter, the CTPA is less posture-dependant and integrates the compensatory mechanisms like pelvic retroversion. A CTPA >20° is considered as sagittal misalignment. Another important point is that T1 Slope Minus Cervical Lordosis (T1S–CL), seems to be the cervical equivalent to PI-LL mismatch, Protopsaltis et al. (2018) identified mismatch >20° as indicative of cervical sagittal deformity in a multicenter retrospective cohort, offering a practical alignment target for surgical planning.

## 5 The impact of cervical sagittal alignment on clinical outcomes

Degenerative cervical myelopathy (DCM) results not only from static compressive pathology—such as spondylosis, osteophytes, or ligamentous hypertrophy—but also from dynamic factors that exacerbate spinal cord injury during flexion and extension (Mohanty et al., 2015; Shamji et al., 2013). Flexion brings the cord against anterior compressive elements; extension increases posterior compression from structures such as the ligamentum flavum (Denaro et al., 2015; Fujiyoshi et al., 2008).

Radiographic parameters such as increased C2–C7 sagittal vertical axis (SVA) and T1 slope (T1S) have consistently been associated with worse clinical outcomes. Smith et al. in a prospective cohort of 56 patients from the AOSpine North America Myelopathy study, demonstrated that increased C2–C7 SVA and C2 slope correlated significantly with lower mJOA scores, underscoring the prognostic relevance of sagittal imbalance (Smith et al., 2013). Additionally, greater T1S – (C2–C7 Cobb angle) mismatch has been linked to worse neurological recovery.

Several reports indicate that patients with preoperative cervical kyphosis exceeding 10° experience worse neurological outcomes^242225^. Uchida et al. (2009) in a comparative study of 96 patients with kyphotic or sigmoid alignment, showed that anterior decompression achieved superior early mJOA recovery and larger postoperative cord cross-sectional area compared with laminoplasty. Shamji et al. (2013) analyzed 124 surgically treated patients and found that preoperative lordotic alignment was associated with greater postoperative neurological recovery, reinforcing the value of sagittal correction. Similarly, Mohanty et al. in a retrospective series of 124 patients, demonstrated that kyphotic alignment was associated with both T2 cord hyperintensity and worse baseline mJOA scores, underscoring deformity-related cord stress (Mohanty et al., 2015).

In cases of ossified posterior longitudinal ligament (OPLL), Fujiyoshi et al. introduced the K-line (Fig. 2), defined by a straight line connecting the midpoints of the spinal canal at C2 and C7 on lateral radiographs. Patients classified as K-line negative (where the OPLL extends beyond the line) had inferior neurological recovery after laminoplasty (Fujiyoshi et al., 2008). Later, Taniyama et al. proposed a modified K-line (mK-line) on MRI, showing that preoperative anterior cord clearance <4 mm was predictive of residual compression and worse outcomes (Shamji et al., 2016; Taniyama et al., 2014).

## 6 Role of surgery in the management of DCM

Prospective studies have confirmed the benefit of surgical intervention in moderate to severe DCM. Surgical decompression halts neurological deterioration and improves function, while nonoperative measures are typically reserved for mildly symptomatic or high-risk patients (Fehlings et al., 2013a, 2013b; Lawrence et al., 2013a).

Although no conclusive evidence favors anterior over posterior surgery for DCM when both approaches are considered options by the operating surgeon, several factors must guide the surgical approach: number and location of compressive levels, sagittal alignment, bone quality, and presence of radiculopathy or neck pain (Fehlings et al., 2012, 2013c; Kato et al., 2017; Nouri et al., 2017; Lawrence et al., 2013b).

In general, anterior decompression is preferred for patients with anterior compression, kyphosis, or multilevel disc herniations (but usually not more than 3). Posterior approaches are more suited to multilevel compression, OPLL, and cases with adequate lordosis or congenital stenosis. Kato et al. (2017) used MRI-based propensity-score matching to compare anterior and posterior approaches, finding no significant difference in outcomes after adjusting for baseline alignment and compression patterns. In a separate analysis (Kato et al., 2018), they showed that patients with preoperative cervical deformity presented with more severe disease and tended to have inferior postoperative outcomes. However, long-term outcomes such as reoperation or alignment deterioration remain insufficiently explored.

In a randomized controlled trial, Ghogawala et al. compared 163 patients undergoing anterior vs posterior surgery. There was no significant difference in SF-36 PCS scores at 1 or 2 years, but complication rates were higher in the anterior group (48 % vs 24 %), especially due to dysphagia (Ghogawala et al., 2021). (It is important to note that not all patients were randomized, only patients were randomized for which a group of expert of 15 found that both options were possible.)

## 7 Posterior approach

Posterior decompression—via laminoplasty or laminectomy with instrumented fusion—enlarges the spinal canal and allows the spinal cord to migrate dorsally (Denaro et al., 2015; Tashjian et al., 2009; Epstein, 2003; Baba et al., 1996). Direct decompression of hypertrophied ligamentum flavum and indirect decompression via posterior drift are achieved simultaneously (Denaro et al., 2015).

Standalone laminectomy without fusion is discouraged in patients lacking preoperative lordosis, due to the risk of postoperative kyphosis (Kaptain et al., 2000). Fusion should be considered to maintain or restore alignment (Denaro et al., 2015; Matsunaga et al., 1999; Ryken et al., 2009). Tashjian et al. found that posterior migration of the spinal cord was not significantly influenced by preoperative cervical curvature, thus supporting posterior surgery even in mild kyphosis when fused (Tashjian et al., 2009; Xia et al., 2011).

Moreover, Sielatycki et al. concluded that creating additional lordosis in already lordotic patients did not improve outcomes, suggesting that “any amount of lordosis may be sufficient” (Sielatycki et al., 2016).

Although laminoplasty preserves motion and reduces alteration of biomechanics, it may result in lesser decompression and more postoperative kyphosis compared to laminectomy with fusion (Lee et al., 2020). Lee et al. found no significant difference in long-term clinical outcomes between the two techniques in multilevel DCM.

It is true however that laminectomy without fusion may still be considered for some cases including single level non-junctional decompression or in patients with fixed deformity for whom the primary goal is decompression rather than deformity correction.

## 8 Anterior approach

Anterior decompression is typically indicated in cases with fixed kyphosis >10°, ventral cord compression, or the need for sagittal realignment (Denaro et al., 2015; Uchida et al., 2009; Suda et al., 2003). Interbody devices restore lordosis and enable direct removal of compressive pathology.

Gwinn et al. proposed evaluating “effective lordosis” using a straight-line method between C2 and C7: if anterior osteophytes or disc protrusions extend into this line, true lordosis is considered lost—guiding the surgeon toward anterior correction (Gwinn et al., 2009).

Despite the variability of spinal cord shift across different alignment types, some authors maintain that kyphosis >10° warrants anterior decompression for optimal outcomes (Denaro et al., 2015; Uchida et al., 2009; Suda et al., 2003).

In patients with significant fixed deformity anterior corpectomy of a or more levels may become necessary, but such construct frequently require additional posterior support.

## 9 Impact of global sagittal balance correction on cervical alignment

Spinal alignment should be viewed as an interconnected system. Correction of global sagittal imbalance—such as with lumbar osteotomies—has been shown to result in spontaneous improvements in cervical lordosis (Smith et al., 2013). Manoharan et al. reported that global sagittal correction—such as through lumbar osteotomy—frequently led to reciprocal increases in cervical lordosis, although head center of gravity remained unchanged, implying region-specific compensation (Manoharan et al., 2018).

Interestingly, they also found that the center of gravity of the head (COG) remained independent of global SVA correction, indicating possible compensatory mechanisms specific to the cervical spine.

It has been shown however, that correction of sagittal alignment does not necessary improve outcomes (Kato et al., 2018).

The heterogeneity of study designs and outcome measures precludes pooled statistical analysis; however, the key thresholds and their reported clinical correlations are summarized in Table 2.

**Table 2:** Key findings from the literature on cervical sagittal alignment and clinical outcomes.

Author (Year)	Study Design/N	Parameter(s) Analyzed	Reported Threshold or Mean	Clinical Outcome(s)	Main Conclusion
Smith et al., 2013 (Smith et al., 2013)	Prospective cohort/56 DCM patients	C2–C7 SVA, C2 slope	SVA >40 mm associated with higher disability	Lower mJOA with increasing SVA and C2 slope	Sagittal malalignment predicts worse neurological function
Oe et al., 2015 (Oe et al., 2015)	Cross-sectional/> 500 asymptomatic volunteers >50 yrs	T1 Slope, CL, T1S–CL mismatch	T1S < 40°, CL > 15°, T1S–CL < 20° identified as normative	Higher T1S–CL mismatch linked to lower EQ-5D	Established age-adjusted normal ranges and correlation with HrQoL
Protopsaltis et al., 2018 (Protopsaltis et al., 2018)	Multicenter retrospective/thoracolumbar osteotomy cohort	T1S–CL mismatch	Mismatch >20° defined cervical sagittal deformity	Larger mismatch linked to worse outcomes after osteotomy	T1S–CL is a practical surgical-planning metric
Uchida et al., 2009 (Uchida et al., 2009)	Comparative cohort/96 DCM with kyphosis or sigmoid alignment	Cervical kyphosis angle, surgical approach	Kyphosis >10° patients benefited more from anterior decompression	Better early mJOA gain and larger postoperative cord CSA	Kyphotic alignment requires anterior correction for optimal decompression
Mohanty et al., 2015 (Mohanty et al., 2015)	Retrospective/124 DCM patients	Cervical alignment vs MRI T2 cord signal	Kyphotic alignment associated with cord T2 hyperintensity	Worse baseline mJOA in kyphotic group	Alignment deformity linked with structural cord injury and worse severity
Shamji et al., 2016 (Shamji et al., 2016)	Prospective cohort/124 surgically-treated DCM	Pre-op lordosis vs neurological recovery	Pre-op lordosis vs kyphosis	Lordotic patients had higher postoperative mJOA recovery	Pre-operative lordosis predicts better neurological recovery
Fujiyoshi et al., 2008 (Fujiyoshi et al., 2008)/Taniyama et al., 2014 (Taniyama et al., 2014)	Retrospective imaging studies/OPLL patients	K-line and mK-line on MRI	mK-line clearance <4 mm predicts residual compression	Poorer mJOA improvement after laminoplasty when K-line negative	K-line guides approach selection and predicts decompression success
Manoharan et al., 2018 (Manoharan et al., 2018)	Retrospective/global-sagittal-correction cohort	Global SVA vs cervical curvature	Post-lumbar correction improved cervical lordosis	Head COG unchanged	Demonstrates reciprocal cervical change after global correction
Kato et al., 2017 (Kato et al., 2017)**/2018** (Kato et al., 2018)	MRI-based propensity-score-matched analysis/large multicenter cohorts	Alignment, surgical approach (ant vs post)	No significant outcome difference after matching for alignment	Worse baseline disease with cervical deformity	Alignment strongly affects baseline severity; approach choice neutral after adjustment
Ghogawala et al., 2021 (Ghogawala et al., 2021)	Randomized controlled trial/163 CSM patients	Surgical approach vs outcomes	–	No SF-36 PCS difference at 1–2 yrs; higher dysphagia in anterior group	Approach choice guided by anatomy/alignment; similar long-term outcomes

## 10 Strength and limitations of current evidence

Most studies linking sagittal parameters such as C2–C7 SVA and T1S-CL mismatch to neurological or functional outcomes are retrospective cohort or case-control studies (Level III evidence) with relatively small to moderate sample sizes and considerable heterogeneity in radiographic techniques and outcome measures. While several analyses demonstrate moderate correlations between increased SVA or higher T1S-CL mismatch and worse mJOA or EQ-5D scores, the lack of prospective randomized data limits causal inference. Furthermore, variability in cutoff thresholds across studies (e.g., SVA >40 mm or T1S-CL >20°) reflects the absence of standardized definitions for cervical sagittal deformity. These limitations should be considered when interpreting the prognostic value of these parameters in surgical planning.

The majority of available data originate from retrospective cohort studies (Level III) or case series (Level IV), with only a limited number of prospective comparative studies and an absence of randomized controlled trials (Level I). This level of evidence underscores the need for multicenter prospective studies to better establish standardized cut-off values and their true prognostic significance.

## 11 Future directions

Establishing robust corrective thresholds will require prospective multicenter cohort studies using standardized radiographic protocols and harmonized outcome reporting. Future research should also evaluate the contribution of dynamic imaging (e.g., upright MRI, flexion–extension radiographs) to detect functional cervical deformity that static imaging may miss. Additionally, incorporating patient-reported outcomes such as neck disability indices, health-related quality of life metrics measures will be essential to link radiographic correction with meaningful functional improvement.

## 12 Illustrative case

**Fig. 4:**
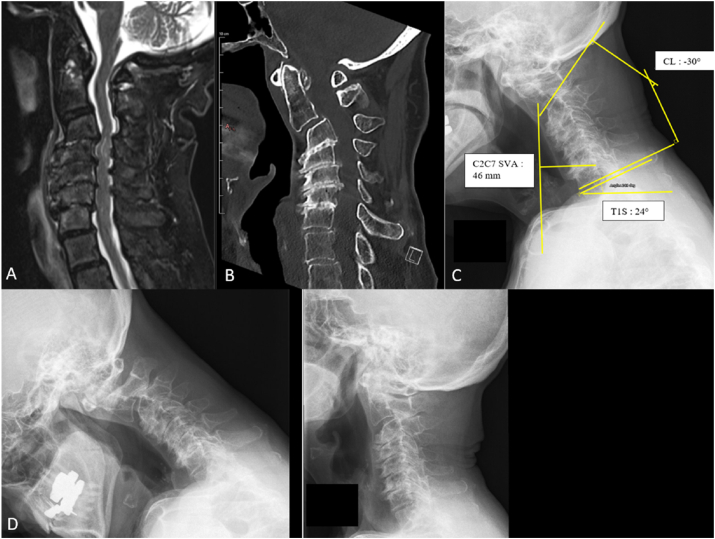
Pre operative MRI (T2 Sagittal- A) CT scan (B) and Standing Radiographs (C) showing preoperative sagittal alignment parameters. Here, the SVA is estimated at 46 mm, indicative of cervical malalignment in the context of extreme cervical kyphosis (−30° for the CL angle) with a T1S – CL mismatch of 54°. Dynamic radiographs (D) reveals the microinstability of the segments C2 to C4.

**Fig. 5:**
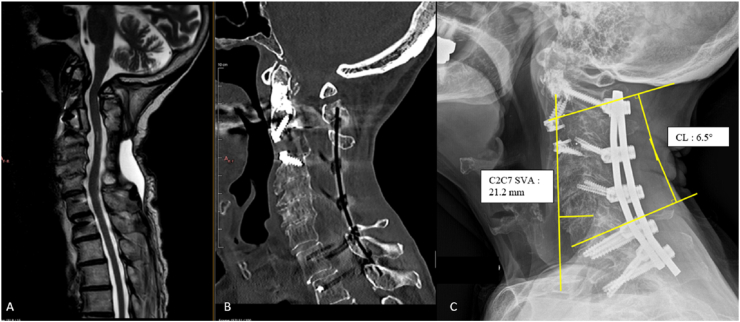
Post-operative sagittal T2 MRI (A) showing the adequate decompression of the spinal cord. CT scan (B) and standing radiographs (C) illustrating the appropriate position of the material with normalization of SVA (<40 mm) and restauration of a cervical lordosis of 6.5°.

An 82-year-old woman with severe osteoporosis, degenerative spine disease, and a prior ischemic stroke presented with progressive gait disturbance, frequent falls, cervical postural deformity (“dropped head”), paresthesia, and difficulty maintaining horizontal gaze. Neurological examination showed bilateral hand weakness (M4), proprioceptive deficits, and an unsteady gait with a positive Romberg sign; mJOA was calculated at 12 (moderate myelopathy), NDI of 15. Imaging revealed fixed cervical kyphosis (CL = −30°) with C2–C7 SVA = 46 mm and T1S–CL mismatch **= 54°**, multilevel compression (C3–C7) with T2 hyperintensity, and C2–C4 micro-instability on dynamic radiographs (Fig. 4). Given the severe fixed kyphosis (>10°) and marked T1S–CL mismatch, a combined anterior–posterior approach was planned to achieve both decompression and sagittal realignment.Surgery involved C3 corpectomy with a 19-mm expandable cage, cement augmentation of the odontoid, followed by C4–C7 laminectomy and C2–D2 posterior fixation with fusion (Fig. 5).The postoperative course was uneventful; the patient was discharged on day 8. At 1-year follow-up, she showed significant functional improvement (mJOA = 15, NDI = 5), restoration of cervical lordosis (CL = 6.5°) and normalized SVA <40 **mm** with stable instrumentation. Residual mild shoulder stiffness was managed conservatively.

## 13 Conclusion

The association of DCM with sagittal cervical imbalance in kyphosis appears to contribute to worse neurological outcomes. Although current evidence is predominantly Class II–III and largely observational, it suggests that addressing sagittal imbalance during surgical planning may improve clinical outcomes. Therefore, in patients with fixed kyphotic deformities exceeding 10°, posterior decompression alone should be approached cautiously, and anterior or combined approaches may be considered on a case-by-case basis. Measurement techniques such as effective cervical lordosis remain practical tools to help guide individualized surgical strategy.

## Informed consent

Informed consent for publication was obtained from the patient for the illustratitve case, and all identifying information has been anonymized.

## Declaration of competing interest

The authors declare that they have no known competing financial interests or personal relationships that could have appeared to influence the work reported in this paper.

## References

[bib1] Albert T.J., Vacarro A. (1998). Postlaminectomy kyphosis. Spine..

[bib2] Ames C.P., Blondel B., Scheer J.K. (2013). Cervical radiographical alignment: comprehensive assessment techniques and potential importance in cervical myelopathy. Spine..

[bib3] Baba H., Uchida K., Maezawa Y., Furusawa N., Azuchi M., Imura S. (1996). Lordotic alignment and posterior migration of the spinal cord following en bloc open-door laminoplasty for cervical myelopathy: a magnetic resonance imaging study. J. Neurol..

[bib4] Buell T.J., Buchholz A.L., Quinn J.C., Shaffrey C.I., Smith J.S. (2018). Importance of sagittal alignment of the cervical spine in the management of degenerative cervical myelopathy. Neurosurg. Clin..

[bib5] Cho K.J., Bridwell K.H., Lenke L.G., Berra A., Baldus C. (2005). Comparison of smith-petersen versus pedicle subtraction osteotomy for the correction of fixed sagittal imbalance. Spine..

[bib6] Denaro V., Longo U.G., Berton A., Salvatore G., Denaro L. (2015). Cervical spondylotic myelopathy: the relevance of the spinal cord back shift after posterior multilevel decompression. A systematic review. Eur. Spine J..

[bib7] Diebo B.G., Challier V., Henry J.K. (2016). Predicting cervical alignment required to maintain horizontal gaze based on global spinal alignment. Spine..

[bib8] Epstein N.E. (2003). Laminectomy for cervical myelopathy. Spinal Cord..

[bib9] Fehlings M.G., Smith J.S., Kopjar B. (2012). Perioperative and delayed complications associated with the surgical treatment of cervical spondylotic myelopathy based on 302 patients from the AOSpine North America cervical spondylotic myelopathy study. J. Neurosurg. Spine..

[bib10] Fehlings M.G., Wilson J.R., Yoon S.T., Rhee J.M., Shamji M.F., Lawrence B.D. (2013). Symptomatic progression of cervical myelopathy and the role of nonsurgical management: a consensus statement. Spine..

[bib11] Fehlings M.G., Wilson J.R., Kopjar B. (2013). Efficacy and safety of surgical decompression in patients with cervical spondylotic myelopathy: results of the AOSpine North America prospective multi-center study. J Bone Joint Surg Am..

[bib12] Fehlings M.G., Barry S., Kopjar B. (2013). Anterior versus posterior surgical approaches to treat cervical spondylotic myelopathy: outcomes of the prospective multicenter AOSpine North America CSM study in 264 patients. Spine..

[bib13] Fujiyoshi T., Yamazaki M., Kawabe J. (2008). A new concept for making decisions regarding the surgical approach for cervical ossification of the posterior longitudinal ligament: the K-line. Spine..

[bib14] Ghogawala Z., Terrin N., Dunbar M.R. (2021). Effect of ventral vs dorsal spinal surgery on patient-reported physical functioning in patients with cervical spondylotic myelopathy: a randomized clinical trial. JAMA..

[bib15] Girod P.P., Lener S., Kögl N. (2023). Health-related quality of life 2 years after pedicle subtraction osteotomy for sagittal imbalance: a single-center experience of 65 patients. Acta Neurochir..

[bib16] Gwinn D.E., Iannotti C.A., Benzel E.C., Steinmetz M.P. (2009). Effective lordosis: analysis of sagittal spinal canal alignment in cervical spondylotic myelopathy. J. Neurosurg. Spine..

[bib17] Iyer S., Lenke L.G., Nemani V.M. (2016). Variations in occipitocervical and cervicothoracic alignment parameters based on age: a prospective study of asymptomatic volunteers using full-body radiographs. Spine..

[bib18] Kaptain G.J., Simmons N.E., Replogle R.E., Pobereskin L. (2000). Incidence and outcome of kyphotic deformity following laminectomy for cervical spondylotic myelopathy. J. Neurosurg..

[bib19] Kato S., Nouri A., Wu D., Nori S., Tetreault L., Fehlings M.G. (2017). Comparison of anterior and posterior surgery for degenerative cervical myelopathy: an MRI-based propensity-score-matched analysis using data from the prospective multicenter AOSpine CSM North America and international studies. J Bone Joint Surg Am..

[bib20] Kato S., Nouri A., Wu D., Nori S., Tetreault L., Fehlings M.G. (2018). Impact of cervical spine deformity on preoperative disease severity and postoperative outcomes following fusion surgery for degenerative cervical myelopathy: sub-Analysis of AOSpine North America and international studies. Spine..

[bib21] Kim K.T., Lee S.H., Suk K.S., Lee J.H., Jeong B.O. (2012). Outcome of pedicle subtraction osteotomies for fixed sagittal imbalance of multiple etiologies: a retrospective review of 140 patients. Spine.

[bib22] Lawrence B.D., Shamji M.F., Traynelis V.C. (2013). Surgical management of degenerative cervical myelopathy: a consensus statement. Spine.

[bib23] Lawrence B.D., Jacobs W.B., Norvell D.C., Hermsmeyer J.T., Chapman J.R., Brodke D.S. (2013). Anterior versus posterior approach for treatment of cervical spondylotic myelopathy: a systematic review. Spine.

[bib24] Le Huec J.C., Faundez A., Dominguez D., Hoffmeyer P., Aunoble S. (2015). Evidence showing the relationship between sagittal balance and clinical outcomes in surgical treatment of degenerative spinal diseases: a literature review. Int. Orthop..

[bib25] Lee S.H., Hyun S.J., Jain A. (2020). Cervical sagittal alignment: literature review and future directions. Neurospine..

[bib26] Liu S., Lafage R., Smith J.S. (2015). Impact of dynamic alignment, motion, and center of rotation on myelopathy grade and regional disability in cervical spondylotic myelopathy. J. Neurosurg. Spine..

[bib27] Lopez A.J., Scheer J.K., Leibl K.E., Smith Z.A., Dlouhy B.J., Dahdaleh N.S. (2015). Anatomy and biomechanics of the craniovertebral junction. Neurosurg. Focus.

[bib28] Manoharan S.R., Joshi D., Owen M., Theiss S.M., Deinlein D. (2018). Relationship of cervical sagittal vertical alignment after sagittal balance correction in adult spinal deformity: a retrospective radiographic study. Int. J. Spine Surg..

[bib29] Matsunaga S., Sakou T., Nakanisi K. (1999). Analysis of the cervical spine alignment following laminoplasty and laminectomy. Spinal Cord..

[bib30] Mohanty C., Massicotte E.M., Fehlings M.G., Shamji M.F. (2015). Association of preoperative cervical spine alignment with spinal cord magnetic resonance imaging hyperintensity and myelopathy severity: analysis of a series of 124 cases. Spine..

[bib31] Nouri A., Tetreault L., Singh A., Karadimas S.K., Fehlings M.G. (2015). Degenerative cervical myelopathy: epidemiology, genetics, and pathogenesis. Spine..

[bib32] Nouri A., Martin A.R., Nater A. (2017). Influence of magnetic resonance imaging features on surgical decision-making in degenerative cervical myelopathy: results from a global survey of AOSpine international members. World Neurosurg..

[bib33] Oe S., Togawa D., Nakai K. (2015). The influence of age and sex on cervical spinal alignment among volunteers aged over 50. Spine.

[bib34] Protopsaltis T., Terran J., Soroceanu A. (2018). T1 slope minus cervical lordosis (TS-CL), the cervical answer to PI-LL, defines cervical sagittal deformity in patients undergoing thoracolumbar osteotomy. Int. J. Spine Surg..

[bib35] Ryken T.C., Heary R.F., Matz P.G. (2009). Cervical laminectomy for the treatment of cervical degenerative myelopathy. J. Neurosurg. Spine.

[bib36] Scheer J.K., Tang J.A., Smith J.S. (2013). Cervical spine alignment, sagittal deformity, and clinical implications: a review. J. Neurosurg. Spine.

[bib37] Scheer J.K., Ames C.P., Deviren V. (2013). Assessment and treatment of cervical deformity. Neurosurg. Clin..

[bib38] Shamji M.F., Ames C.P., Smith J.S., Rhee J.M., Chapman J.R., Fehlings M.G. (2013). Myelopathy and spinal deformity: relevance of spinal alignment in planning surgical intervention for degenerative cervical myelopathy. Spine.

[bib39] Shamji M.F., Mohanty C., Massicotte E.M., Fehlings M.G. (2016). The association of cervical spine alignment with neurologic recovery in a prospective cohort of patients with surgical myelopathy: analysis of a series of 124 cases. World Neurosurg..

[bib40] Sielatycki J.A., Armaghani S., Silverberg A., McGirt M.J., Devin C.J., O'Neill K. (2016). Is more lordosis associated with improved outcomes in cervical laminectomy and fusion when baseline alignment is lordotic?. Spine J..

[bib41] Smith J.S., Lafage V., Ryan D.J. (2013). Association of myelopathy scores with cervical sagittal balance and normalized spinal cord volume: analysis of 56 preoperative cases from the AOSpine North America myelopathy study. Spine..

[bib42] Suda K., Abumi K., Ito M., Shono Y., Kaneda K., Fujiya M. (2003). Local kyphosis reduces surgical outcomes of expansive open-door laminoplasty for cervical spondylotic myelopathy. Spine.

[bib43] Taniyama T., Hirai T., Yoshii T. (2014). Modified K-line in magnetic resonance imaging predicts clinical outcome in patients with nonlordotic alignment after laminoplasty for cervical spondylotic myelopathy. Spine..

[bib44] Tashjian V.S., Kohan E., McArthur D.L., Holly L.T. (2009). The relationship between preoperative cervical alignment and postoperative spinal cord drift after decompressive laminectomy and arthrodesis for cervical spondylotic myelopathy. Surg. Neurol..

[bib45] Uchida K., Nakajima H., Sato R. (2009). Cervical spondylotic myelopathy associated with kyphosis or sagittal sigmoid alignment: outcome after anterior or posterior decompression. J. Neurosurg. Spine..

[bib46] Williamson T.K., Krol O., Tretiakov P. (2023). Crossing the bridge from degeneration to deformity: when does sagittal correction impact outcomes in adult spinal deformity surgery?. Spine.

[bib47] Xia G., Tian R., Xu T., Li H., Zhang X. (2011). Spinal posterior movement after posterior cervical decompression surgery: clinical findings and factors affecting postoperative functional recovery. Orthopedics..

